# Autism Spectrum Disorder in a Girl with a *De Novo* X;19 Balanced Translocation

**DOI:** 10.1155/2012/578018

**Published:** 2012-05-17

**Authors:** Marcelo Razera Baruffi, Deise Helena de Souza, Rosana Aparecida Bicudo da Silva, Ester Silveira Ramos, Danilo Moretti-Ferreira

**Affiliations:** ^1^Department of Genetics, Bioscience Institute of Botucatu, São Paulo State University (UNESP), 18618-970 Botucatu, SP, Brazil; ^2^Department of Genetics, School of Medicine of Ribeirão Preto, University of São Paulo (USP), 14049-900 Ribeirão Preto, SP, Brazil

## Abstract

Balanced X-autosome translocations are rare, and female carriers are a clinically heterogeneous group of patients, with phenotypically normal women, history of recurrent miscarriage, gonadal dysfunction, X-linked disorders or congenital abnormalities, and/or developmental delay. We investigated a patient with a *de novo* X;19 translocation. The six-year-old girl has been evaluated due to hyperactivity, social interaction impairment, stereotypic and repetitive use of language with echolalia, failure to follow parents/caretakers orders, inconsolable outbursts, and persistent preoccupation with parts of objects. The girl has normal cognitive function. Her measurements are within normal range, and no other abnormalities were found during physical, neurological, or dysmorphological examinations. Conventional cytogenetic analysis showed a *de novo* balanced translocation, with the karyotype 46,X,t(X;19)(p21.2;q13.4). Replication banding showed a clear preference for inactivation of the normal X chromosome. The translocation was confirmed by FISH and Spectral Karyotyping (SKY). Although abnormal phenotypes associated with *de novo* balanced chromosomal rearrangements may be the result of disruption of a gene at one of the breakpoints, submicroscopic deletion or duplication, or a position effect, X; autosomal translocations are associated with additional unique risk factors including X-linked disorders, functional autosomal monosomy, or functional X chromosome disomy resulting from the complex X-inactivation process.

## 1. Introduction

Balanced X-autosome translocations are rare, and female carriers are a clinically heterogeneous group of patients, with phenotypically normal women, history of recurrent miscarriage, gonadal dysfunction, X-linked disorders or congenital abnormalities, and/or developmental delay. As transcriptional silencing can spread into the autosomal chromatin leading to a functional monosomy, there is a preference for a selective inactivation of the normal chromosome [1, 2].

In a collaborative study in association with the UK Association of Clinical Cytogenetics (ACC), a total of 104 women with X-autosome translocation were reported, of whom only four involved chromosome 19 [2]. The patients presented different breakpoints: 46,X,t(X;19)(p11.2;q13.3) *de novo*; 46,X,t(X;19)(q28;p13.3) *de novo*; 46,X,t(X;19)(p11.23;p13); 46,X,t(X;19)(p10;q10) [2].

The present study investigated a patient with a *de novo* X; 19 translocation.

## 2. Patient, Material, and Methods

### 2.1. Patient

**Figure 1:**
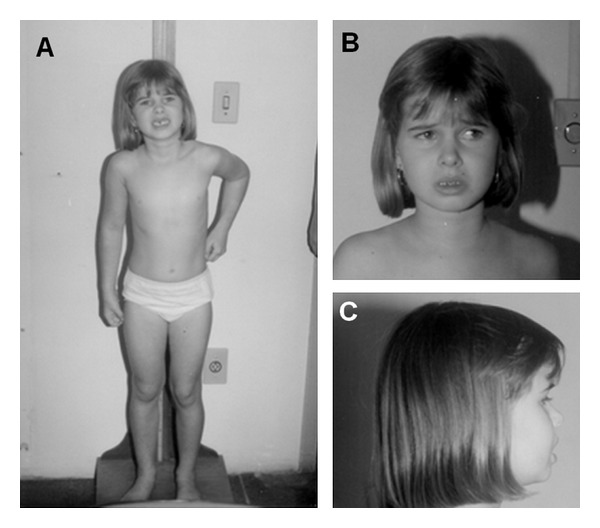
The patient at 6 yo (A), face (B), and profile (C).

The six-year-old girl has been evaluated due to hyperactivity, social interaction impairment, with little social initiative and lack of social or emotional reciprocity, stereotypic and repetitive use of language with echolalia, failure to follow parents/caretakers orders, inconsolable outbursts, and persistent preoccupation with parts of objects. The girl has normal cognitive function. She is the only daughter of a nonconsanguineous young and healthy couple. There are no other similar cases in the family. She was born at term (caesarean delivery), weighing 3800 g and measuring 50.5 cm. She started to walk at 1 year and 4 months, and to speak at 2 years and 6 months. The patient has improved with language and educational therapies. She was examined when she was 6 years and 4 months old, 123 cm (p 50–75) tall, weighing 23,000 g (p 50–75), and had 50.5 cm of head circumference (p 2–50) (Figure 1). The clinical examination showed no physical, neurological, or dysmorphological abnormalities. Hematologic and hormonal test results were normal. The EEG displayed a normal pattern. Bone age was 8 years and 10 months (chronological age was 6 years and 4 months). Brain magnetic resonance imaging was normal. PCR-based assay for sizing CGG repeats at the *FRAXA* locus showed two normal alleles.

### 2.2. Cytogenetic Analysis

#### 2.2.1. Banding

Metaphase chromosome spreads were obtained after lymphocyte temporary cultures, and the slides were subjected to GTG, CBG, and replication bandings.

#### 2.2.2. FISH

 FISH was performed only for verification of the X chromosome with total chromosome digoxigenin-labeled probe obtained commercially (Oncor^r^, Gaithersburg, MD, USA—catalogue no. P5222-DG.5). Hybridization was performed according to manufacturer's instructions, followed by detection with FITC-labeled antidigoxigenin and count stain with propidium iodide (final concentration 0.3 *μ*g/mL in antifade).

#### 2.2.3. Spectral Karyotyping (SKY)

A SKY KIT from Applied Spectral Imaging (ASI, Carlsbad, CA, USA) was used for SKY. The slide treatment, posthybridization detection and washes, was performed as per standard protocols and manufacturer's instructions [3]. The slides were mounted with 20 mM Tris-HCl pH 8.0, 90% glycerol containing 2.3% antifade, 1,4,diazabicyclo-(2.2.2)octane (ONCOR Inc., Gaithersburg, MD, USA). Spectral images were acquired and analyzed with a SD 200 spectral bioimaging system (ASI Ltd., MigdalHaemek, Israel) attached to a Zeiss microscope (Axioplan 2) by means of a C-mount consisting of an optical head with a special Fourier transformed spectrophotometer (SAGNAC common path interferometer) to measure the spectrum, and a refrigerated CCD-camera for imaging. Excitation through the custom filter set (Chroma Technology, Brattleboro, VT, USA) allows all dyes to be excited and measured simultaneously, without any image shift. The generation of a spectral image is achieved by acquiring ~100 frames of the same image. Each two frames differ from each other only in the optical path difference (OPD) created by the scanner controller in the interferometer. The images were stored in a computer for further analysis using the SKYVIEW (ASI, Carlsbad, CA, USA) software. Based on the measurement of the spectrum for each chromosome, a spectral classification algorithm was applied. DAPI images were acquired from all metaphases analyzed using a DAPI-specific optical filter.

## 3. Results

**Figure 2:**
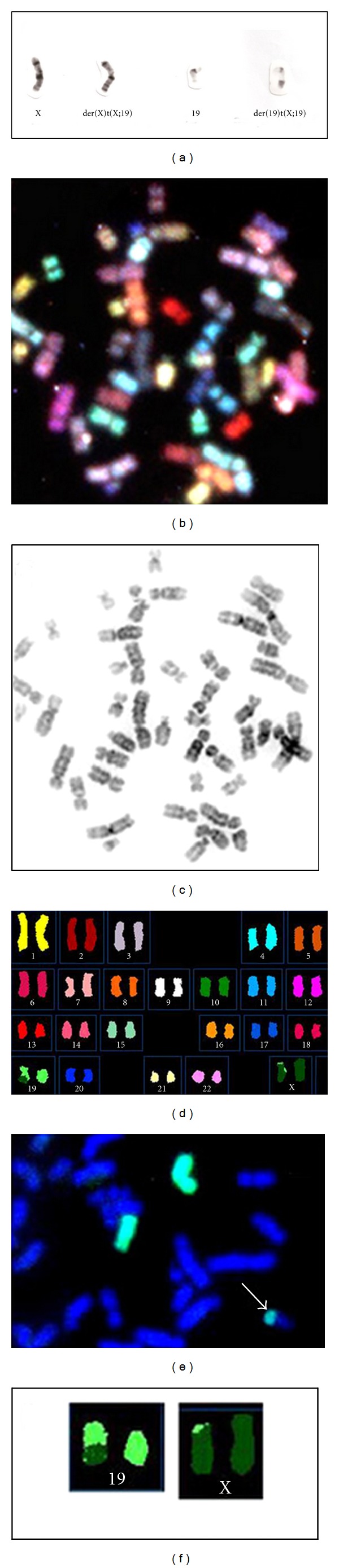
(a) GTG-banded partial metaphases. Chromosomes are (from left to right): normal X, der(X), normal 19, and der(19). (b, c, d, f**) **Spectral Karyotyping (SKY) shows, respectively, the whole metaphase (b), the same metaphase in reverse G banding (c), spectra-based colors following classification (d), and detailed characterization of the abnormal chromosomes (f). (e) FISH using X-chromosome probe.

Conventional GTG banding showed a *de novo* balanced translocation, with the karyotype 46,X,t(X;19)(19qter → 19q13.4::Xp21.3 → Xqter;19pter→19q13.4::Xp21.3→Xpter) (Figure 2). CBG banding revealed a duplication of the 9qh. Additional FISH and SKY analyses confirmed the translocation, as determined by GTG-banding (Figure 2). The normal X chromosome was preferentially inactivated, as shown by replication banding.

## 4. Discussion

Schmidt and Du Sart [4] showed that we might expect to observe translocation breakpoints in distal Xp (Xp22) and Xq (Xq28). However, our patient had a breakpoint in Xp21.2. This is in agreement with another study that showed an apparently random breakpoint distribution, except for overrepresentation at Xq22 [2].

Armah et al. [5] described a case of a translocation involving chromosomes X and 19, t(X;19)(p11.2;q13.1) in a renal cell carcinoma (RCC) occurring during pregnancy. There is an association between Xp11.2 translocations and RCC. There is another report of a hepatic desmoplastic tumour with translocation (X;19)(q13;q13.3) [6]. Our patient presented distinct Xp and 19q breakpoints.

In another study very similar to the present one, a patient with a balanced reciprocal translocation 46,XX,t(X;19)(p21;q13) had associated features including short stature, unilateral kidney hypoplasia, a brachial cyst, and Diamond-Blackfan anaemia (DBA) [1]. The latter is a rare pure red-cell hypoplasia, and a DBA locus has been localized to chromosome 19q13.2. The 19q13.4 breakpoint could explain the absence of the anaemia in our patient.

Dystrophin gene is mapped on Xp21.2-p21.1. This gene and dystrophinopathies have been associated with autism [7]. No muscular disorder was evident in the present study.

Autism is also linked with cytogenetic abnormalities found at the 15q1-q13 locus, and many case reports have described duplications, deletions, and inversions at this locus [8]. Muhle et al. [8] suggested that the *γ*-amino butyric acid (GABA) receptor gene cluster (located at 15q11-q13) is strongly implicated in the pathogenesis of autism, given its involvement in the inhibition of excitatory neural pathways. In another study, an association analysis was performed for a marker of GABRB3-called 155CA-2, using the transmission disequilibrium test (TDT) in a set of 80 autism families [9]. These authors suggested that genetic variants within the GABA receptor gene complex in 15q11-q13 may play a role in autistic disorder.

The patient in our case may have undergone a submicroscopic deletion on the X or chromosome 9 at the breakpoints. Epigenetic and/or position effect cannot be ruled out either. The skewed X inactivation could allow the lesser amount of clinical features.

Abnormal phenotypes associated with *de novo* balanced chromosomal rearrangements may be the result of disruption of a gene at one of the breakpoints, submicroscopic deletion or duplication, or a position effect, whereas X-autosomal translocations are associated with additional unique risk factors including X-linked disorders, functional autosomal monosomy, or functional X-chromosome disomy resulting from the complex X-inactivation process.
